# Multi Swarm Optimization Based Clustering with Tabu Search in Wireless Sensor Network

**DOI:** 10.3390/s22051736

**Published:** 2022-02-23

**Authors:** Sundararaj Suganthi, Nagappan Umapathi, Miroslav Mahdal, Manickam Ramachandran

**Affiliations:** 1 Department of Computer and Communication, Sri Sairam Institute of Technology, Chennai 600044, India; suganthivasanth81@gmail.com; 2 Department of Electronics and Communication Engineering, Jyothishmathi Institute of Technology and Science, Karimnagar 505481, India; nrumapathi@gmail.com; 3 Department of Control Systems and Instrumentation, Faculty of Mechanical Engineering, VSB-Technical University of Ostrava, 17. Listopadu 2172/15, 708 00 Ostrava, Czech Republic; 4 Data Analytics Lab, REST Labs, Kaveripattinam, Krishnagiri 635112, India

**Keywords:** cluster head (CH), energy consumption, metaheuristics, particle swarm optimization (PSO), wireless energy transfer

## Abstract

Wireless Sensor Networks (WSNs) can be defined as a cluster of sensors with a restricted power supply deployed in a specific area to gather environmental data. One of the most challenging areas of research is to design energy-efficient data gathering algorithms in large-scale WSNs, as each sensor node, in general, has limited energy resources. Literature review shows that with regards to energy saving, clustering-based techniques for data gathering are quite effective. Moreover, cluster head (CH) optimization is a non-deterministic polynomial (NP) hard problem. Both the lifespan of the network and its energy efficiency are improved by choosing the optimal path in routing. The technique put forth in this paper is based on multi swarm optimization (MSO) (i.e., multi-PSO) together with Tabu search (TS) techniques. Efficient CHs are chosen by the proposed system, which increases the optimization of routing and life of the network. The obtained results show that the MSO-Tabu approach has a 14%, 5%, 11%, and 4% higher number of clusters and a 20%, 6%, 14%, and 6% lesser average packet loss rate as compared to a genetic algorithm (GA), differential evolution (DE), Tabu, and MSO based clustering, respectively. Moreover, the MSO-Tabu approach has 136%, 36%, 136%, and 38% higher lifetime computation, and 22%, 16%, 51%, and 12% higher average dissipated energy. Thus, the study’s outcome shows that the proposed MSO-Tabu is efficient, as it enhances the number of clusters formed, average energy dissipated, lifetime computation, and there is a decrease in mean packet loss and end-to-end delay.

## 1. Introduction

Wireless Sensor Networks (WSNs) can be defined as a network of many cost-effective, efficient, and multifunctional sensor nodes (SNs) working as a package to monitor an area of interest (AoI). The sensors gather data from the AoI and relay them back to a base station (BS), where it is processed. Wireless Sensor Networks (WSNs) have helped monitor remote environments. It is very effective in collecting data in several inaccessible areas, such as coastguard, forestry, war-prone areas, underwater study, climatic changes, etc. Many sensor nodes are present in WSNs, which are linked with one another and also to a base station. In remote areas, it is impossible to charge and reinstall sensor nodes [1,2]. There are several challenges and restrictions in WSNs, such as: (1) The prime concern is energy as the powerhouse for WSNs are batteries which are bound by time constraints; (2) The next major challenge is self-management as WSNs usually operate in inaccessible and rough environments; (3) Several challenges including mitigation in the medium of propagation and increased distance between SNs and BS are major challenges faced by WSNs; (4) In WSN, there is design constrain as many desirable components are prohibited by the integration because of energy, storage, and other difficulties and so a big challenge in energy conservation. Researchers have resolved this issue more recently by forming clusters from nodes, thus improving its lifespan [3,4].

The network’s lifetime is increased by monitoring regions classified into several regions called clusters. Many sensor nodes are found in each cluster. Only one sensor operates as the cluster head (CH), while the remaining work as cluster members. The information is collected from respective sensors in the specified region. During the selection of CH in every region, the node with the increased energy residue compared to other nodes is chosen as CH [5].

To enhance the network’s lifetime, the most crucial part is the selection of the CH. The various steps for this can be grouped as (i) node with maximum power residue, (ii) rotating CH at specific interludes, (iii) CH selection based on the rotation of communication among the sink and CH, (iv) algorithms for optimized CH selection. In cluster WSNs, implementation of routing protocols is done to provide guidance to the choice of CHs and locate the best passage that can save the node’s energy.

When the network has multiple clusters, it is called clustering routing; every cluster has a tree structure that consists of several CH and cluster members. The information gathered by cluster members is passed on to CH to be compiled through multi-hop CHs or directly passed on to the BS. The outcome of the clustering routing algorithm in each round of CH selection benefits energy load balancing of the network node; the data that is collected by the member nodes of the cluster is forwarded by CH after a merger to mitigate the energy consumed by the network to transmit data and avoid every cluster member node that forwards data individually [6,7]. For WSNs, clustering routing algorithms are widely used; the classic clustering routing algorithms such as Low Energy Adaptive Clustering Hierarchy (LEACH), Hybrid Energy Efficient Distributed clustering protocol (HEED), and other agreements.

The issue of energy-balanced selection of CH is NP-hard. Many detailed reviews on these are available in the literature [8,9]. Literature has put forth many heuristic and metaheuristic clustering algorithms, but these suffer from an imbalance in energy consumption [10]. In this issue, there is no uniform distribution of CHs, while highly loaded CHs perish faster than under-loaded CHs. This is because of the idea that the average distance between CH and BS is utilized in the fitness function, which selects all CHs closer to BS. This problem is solved in this paper by multi swarm optimization (MSO) with a Tabu search (TS) algorithm based on clustering in WSN. The proposed method in this paper improves energy efficiency and efficiency. The rest of the paper is organized into the following sections. Works related to literature are discussed in Section 2. The methodology is discussed in Section 3. Experimental results are discussed in Section 4, and Section 5 concludes the work.

## 2. Related Works

When there is an imbalance in the transferring information in the communication channel, an energy hole is created, causing the premature death of the SNs, which decreases the network’s lifetime [11,12]. This problem was resolved by Krishnan et al. [13,14], who proposed an approach in which the transmission range was adjusted based on the distance between CHs and their members. Also, the lifetime of the network was increased by the application of the firefly optimization algorithm. This algorithm put forth was compared with standardized algorithms under several situations. The outcome of their study showed that this algorithm showed better results concerning the life of the network. However, the author does not address the SNs’ overall energy maximization while maintaining the ideal CH distance.

Three objectives were put forth by Xu et al. [15] to tackle the coverage problem in WSN optimization to balance between the lifetime of the network and coverage. These minimize the consumption of power and rate of coverage while maximizing its balance. Two developed hybrid multi-objective evolutionary algorithms based on decomposition, namely hybrid-MOEA/D-I and hybrid-MOEA/D-II, have been put forth; using the famous multi-objective evolutionary algorithm on decomposition (MOEA/D), hybrid-MOEA/D-I, hybrid genetic algorithm (GA), and a differential evolutionary (DE) algorithm which can efficiently optimize the coined multi-objective optimization problem. With the integration of discrete particle swarm algorithm, it was found to further improve solutions created using Hybrid-MOEA/D-I in a new Hybrid-MOEA/D-II algorithm, as MOEA is not a computationally efficient algorithm and not suitable for balancing the SN’s energy and coverage. Thus to maximize the energy efficiency of the SN, the controlled algorithm should be computationally efficient. Thus we are motivated to develop a computationally efficient hybrid algorithm for getting the said solution in the stipulated time frame.

PSO-UFC (PSO-based Unequal and Fault tolerant Clustering protocol) was put forth by Kaur and Kumar [16]. Imbalanced clustering and fault tolerance issues in the current Energy-Balanced Unequal Clustering (EBUC) technique were addressed in the technique proposed for the effective functioning of the network over the long run. To solve the imbalance in a clustering problem, a lack of inequality in the clustering mechanism was utilized to poise intra- and inter-cluster energy utilization among the Master CHs (MCHs). Furthermore, in the PSO-UFC protocol, there was a restoration of network connectivity through the choice of an extra CH called Surrogate CH because of an impulsive MCH failure. However, it is observed that the lifetime maximization of SNs depends on the optimized clustering strategy while reducing end-to-end delay. Thus, the motivation is to develop an optimal clustering technique with minimal packet loss and end-to-end delay to meet the essential requirement towards maximizing the SNs lifetime.

Regional Energy Aware Clustering with Isolated Nodes (REAC-IN) was presented by Varsha et al. [17] for clustering. The choice of CH is done in REAC-In by weight and weight, which is measured through residual energy of every sensor and the mean regional energy of entire sensors in the individual cluster. Good results were shown by this technique compared to the available WSN protocols, but many issues have been neglected. To get over these restrictions, a new, improved technique was proposed. It was shown that the proposed technique could overcome the constraints of the REAC-IN routing technique through the use of clustering and TS. The available techniques were outperformed by their proposed technique. The issue of the clustering algorithm’s complexity, which is a critical component in the views of maximizing the lifetime of SNs, is still unaddressed.

An enhanced Cuckoo Search (CS) based energy balanced node clustering protocol was put forth by Gupta [18] using a new objective function that involves the uniform distribution of CHs. In another work, Gupta and Jha [19] used an enhanced Harmony Search (HS) based routing protocol is presented to route the data packet among CHs and the sink. The average energy consumption, the number of live and dead nodes, and network lifetime were used to evaluate the effectiveness of the presented integrated clustering and routing protocol. When contrasted over the state-of-the-art protocols, the new Cuckoo-Harmony Search-based integrated routing and clustering protocol explained greater outcomes. The author neglects to include the lifetime and end-to-end delay components while formulating the state-of-the-art meta-heuristic protocols with integrated routing and clustering. As a result, the focus of this research is on clustering of SNs with optimal network lifetimes to leverage optimal coverage.

Similarly, Fish Swarm Optimization (FSO) based multi-hop clustering algorithm to conserve WSN energy utilization was presented by Shanthi and Sundarambal [20]. The result shows that the proposed method performs better than the conventional models. Consequently, Marhoon et al. [21] developed a novel algorithm to increase the efficacy of the LEACH protocol for improving the clustering goal. Thereafter, Marhoon and Awaad [22] proposed an improved version of the LEACH protocol to reduce the energy consumption in WSNs. However, the network lifetime maximization under the constraint of effective packet delivery in a minimum end-to-end delay framework is not included in the literature investigations. Thus, we are motivated to find the solutions for the shortfall in the literature studies with the development of an efficient meta-heuristic algorithm.

## 3. Methodology

PSO, an algorithm based on swarm intelligence, gains its motivation from the social behavior of a flock of birds, a school of fish, etc., generating optimal solutions. Swarm is the population of particles in PSO. An optimal solution is reached by the flying of particles that are theoretical entities through search space. Multiple probable solutions are maintained simultaneously by PSO. In this section, TS, MSO based clustering, and MSO with TS methods are discussed. Tabu search [23,24,25] and PSO [26,27,28] have found some applications in WSN optimization.

### 3.1. Tabu Search (TS) Algorithm

A local heuristic search that can discover the solution space ahead of optimality is guided through the metaheuristic TS. A TS strategy is employed that goes beyond local search adapting N(*x*) as the search develops, by effective replacement through another neighborhood N*(*x*). The move is the operation through which a local or neighborhood search process is guided to attain the solution *x*′ ∈ N(*x*) from the solution *x* ∈ X iteratively and is associated with the neighborhood of solution *x*. TS moves towards its closest neighbor at every iteration, even if the objection function deteriorates. This is different from the classical hill-climbing process as it is only permitted to move to the neighbor solution with improvement objective function [29,30].

Special memory structures are used, which is the prime feature of the TS approach that serves in determining N*(*x*), and it is organized in the manner where exploration of space is done. To avoid cycling, certain moves are prohibited by TS from being re-instantiated for a specific period by utilizing a special memory structure that is short-termed called recency-based memory implemented through the Tabu list. By assigning Tabu-active designation, recency-based memory is exploited to choose attributes that take place in Tabu-active designation. Tabu-tenure is the iteration span to which an attributed is maintained as Tabu. Additionally, aspiration criteria are used by TS as a device in overriding the Tabu status of a move, thus offering a flexible performance. TS protocol’s pseudocode is shown below in Algorithm 1 [29,30].
**Algorithm 1** TS protocol’s pseudocode1.
Start
2.
Select An initial x∈X
3.
$Let\;x^{*}:=x.\left\{x^{*}denote\;the\;best\;soultion\;currently\;founded\right\}$
4.
Set the iteration number k=0.
5.
$T=\phi .\;\left\{T\;is\;Tabu\;List\right\}$
6.
$If\;S\left(x\right)-T\;is\;empty$
7.
go to step 16.
8.
Else
9.
k=k+1
10.
$Select\;x_{k}\in S\left(x\right)-T\;such\;that\;$

$s_{k}\left(x\right)\in Optimum\left(s\left(x\right):s\in S\left(x\right)-T\right)$
11.
End it
12.
$x:=s_{k}\left(x\right)$
13.
$If\;C\left(x\right)<C\left(x^{*}\right)$
14.
$x^{*}:=x$
15.
End if
16.
If a chosen number of iterations has elapsed either in total 

$or\;sin\;c\;e\;x*was\;last\;improved,\;or\;if\;S\left(x\right)-T=\phi$
17.
Upon reaching this step directly from step 6.
18.
Else
19.
$Update\;T\;\left(as\;subsequently\;identified\right)$
20.
Return to step 6.
21.
End if.
22.
End.


### 3.2. Multi Swarm Optimization Based Clustering (MSO)

This work discusses the multi-PSO swarm, which does not generalize instantly as there is no interaction between the swarm. There might be interaction if it is right to attract another swarm; however, one swarm decreases through a number of information sharing topologies. Two multi-swarm methods are suggested here. Multi-Charged Particle Swarm Optimization (CPSO) is a multi-population variant of CPSO. Multi-Quantum Swarm Optimization (MQSO) exploits quantum swarms based on quantum in place of traditional atom objectives.

Blackwell introduced CPSO [31] with the basis of the orbit model of a nucleus atom that is orbited by electrons. In CPSO, the charge is given to a certain proportion of particles that repel other charged particles. Charged particles are those given a charge, whereas the others are termed neutral particles. Vanilla update equation is used to update particle velocities with an additional term based on their proximity to other particles.

(1) $$\vec{v_{i}}\left(t+1\right)=w\vec{v_{i}}\left(t\right)+c_{1}\vec{r_{1}}\left(t\right)(\vec{pbest}-\vec{x_{i}}\left(t\right))+c_{2}\vec{r_{2}}\left(t\right)(\vec{gbest}-\vec{x_{i}}\left(t\right))$$

In Equation (1), $\vec{v_{i}}$ is the velocity of particle *i*, $\vec{x_{i}}$ is the position of the particle, $\vec{pbest}$ is the personal best position of the particle, and $\vec{gbest}$ is the swarm’s best-found position, assuming a star neighborhood topology. The constants $w\in \left[0,\;1\right],\;c_{1}\geq 0,\;and\;c_{2}\geq 0$ are the inertia, cognitive, and social weights, respectively, which are user-supplied parameters. Inertia applies a portion of the previous velocity to the current velocity in an attempt to keep the direction of the particle from changing. The cognitive and social components verify the involvement obtained from the personal best and global best positions, respectively. At last, $\vec{r_{1}}\left(t\right)$ and $\vec{r_{2}}\left(t\right)$ are vectors sampled from a uniform distribution in the range [0, 1], giving the PSO algorithm its stochastic element.

The CPSO velocity update equation is given as (2):

(2) $$\vec{v_{i}}\left(t+1\right)=w\vec{v_{i}}\left(t\right)+c_{1}\vec{r_{1}}\left(t\right)(\vec{pbest}-\vec{x_{i}}\left(t\right))\;c_{2}\vec{r_{2}}\left(t\right)(\vec{gbest}-\vec{x_{i}}\left(t\right))+\underset{\forall j\neq i}{\sum }a_{ij}$$

The acceleration between particles *i* and *j* is given by (3):

(3) $$a_{ij}=\left\{\begin{array}{c} \frac{Q_{i}Q_{j}}{\left|\left|\vec{x_{i}}-\vec{x_{j}}\right|\right|}(\vec{x_{i}}-\vec{x_{j}})\;if\;p_{core}<\left|\left|\vec{x_{i}}-\vec{x_{j}}\right|\right|\leq p\\ 0\;\;\;\;\;\;\;\;\;\;\;\;\;\;\;\;\;\;\;\;\;\;\;\;\;\;\;\;\;\;\;\;\;\;\;otherwise \end{array}\right.$$

where $Q_{i}$ consigns to the charge of particle i, p and $p_{core}$ control the acceleration term’s radius of effect.

In vanilla PSO, neutral particles react as they would, whereas convergence of neutral particles around gbest will be exhibited by CPSO. However, the diversity loss problem is addressed by the acceleration term during divergence, where charged particles are repelled, causing increased exploration. Thus, a higher level of diversity is designed by CPSO throughout the rut, which eliminates the linear collapse problem that is reminded with vanilla PSO. Even though there is thorough handling of the diversity issue by CPSO, there is the need for an external strategy to address outdated memory [32,33].

Multi-modal dynamic problems are dealt with by the MQSQ algorithm. A swarm is divided into sub swarms to exploit various promising peaks in parallel. Diversity is increased and probability decreased in finalizing the search in local optimum. Moreover, such swarms are made up of two types of particles: (i) PSO particles that follow the standard PSO algorithm but try to reach a better position; (ii) quantum particles that orbit around the sub swarm attractor in a radius $r_{cloud}$ to remain the diversity besides the algorithm implementation. Quantum particles deal with the diversity loss issue [34]. The following equation is used to calculate the position of quantum particles (4):

(4) $$\vec{p_{i}}\in B_{n}\left(r_{cloud}\right)$$

where *B_n_* denotes the d-dimensional ball of the swarm *n* centered on the swarm attractor $\vec{g_{n}}$ with radius $r_{cloud}$.

The objective behind MQSQ is that every swarm has a peak, and the peak is tracked alongside the algorithm implementation. To assure that two swarms are not utilizing the same peak, a swarm interaction is done in an elimination system. An easy competition rule is used by the exclusion mechanism between swarms which are nearby (distance less than $r_{excl}$ from each other). The swarm is the winner with the optimal fitness value at its swarm attractor, while the loser swarm is sent out and reinitialized in the search space.

When the peaks outnumber swarms, the peaks will not be able to track the swarms. Thus, due to altering the environment, other local maximum might be a new global maximum, and at any time its tracking is not done by another swarm there is a decrease in system performance. To avoid this, the application of anti-convergence is done when the swarms converge, i.e., for all swarms, the maximum distance is less than $r_{conv}$. At that moment, the worst swarm is expelled by reinitializing the swarm particles by anti-convergence. Because of this, there is one swarm that looks for fresh peaks.

### 3.3. Proposed MSO-Tabu Algorithm

Instead of a single objective, optimization of a vector of objectives is done in vector optimization of multi-objective optimization (MOO). MOOs are quite helpful in solving a number of multi-objective optimization problems (MOPs), where multiple objects are exploited simultaneously with effect to set of restrictions. However, respective optima cannot be attained through multiple objectives in a similar period. Pure and hybrid TS approaches have been set up through new records to discover the optimal solution to problems in production scheduling and planning, network design, allocation of resources, routing, and so on. The technique chosen for quick solution schemes is the TS scheme in hard combinatorial optimization issues.

There are many advantages with MSO (multi-PSO), which include greater convergence, resolution of optimization effectively, decreased diversity of population, and directing early convergence to a local optimum [35,36]. A potential scholastic optimization technique is TS which could congregate hypothetically symptotically to the global optimum solution; more timespan is needed in achieving the near-global minima. Integrating TS to MSO as a local development method can permit the protocol to maintain the population diversity, avoiding directing towards misguiding local optimum. In TS, average energy utilization is greater than MSO, and the calculation time is less in TS than MSO. Through the merger of both techniques, a compromise between them is avoided. For the hybrid approach, simple MSO and generalized TS are used. The following steps are used and implemented in the proposed algorithm.

- Step 1: Initialization of location and energy of nodes and BS.
- Step 2: Cluster formation take place through computation of the distance between nodes that correspond to the base station and energy level of the nodes.
- Step 3: Verify the optimal local position through MSO.
- Step 4: Find out the optimal global position—the global optimal is computed by initializing the Tabu list with MSO solution and Tabu memory entries to zero. Routes are swapped and create an entry in Tabu memory. The limitations are confirmed.
- Step 5: Find out the subsequent place lest the fitness value exists in the Tabu list, and that fitness value is noted. The optimal solution from the Tabu list with the least hop routing is considered.

## 4. Results and Discussion

In this section, the proposed MSO-Tabu approach is critically evaluated by comparing its performance with GA, DE, Tabu search, and MSO based clustering. The numerical experiments are conducted on a Dell Optiplex^TM^ 3020 personal computer with Intel Core i7-4770 3.4 GHz processor along with 8 MB Cache and integrated Intel graphics, 16 GB RAM, 1 TB HDD, and Windows 10 operating system. Matlab R2019a is used as the simulation environment. The parameters of the network used are reported in Table 1.

**Figure 1:**
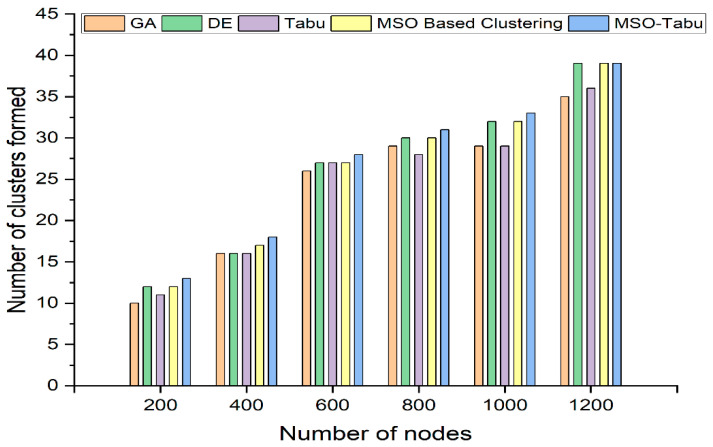
Number of clusters formed for MSO-Tabu.

**Figure 2:**
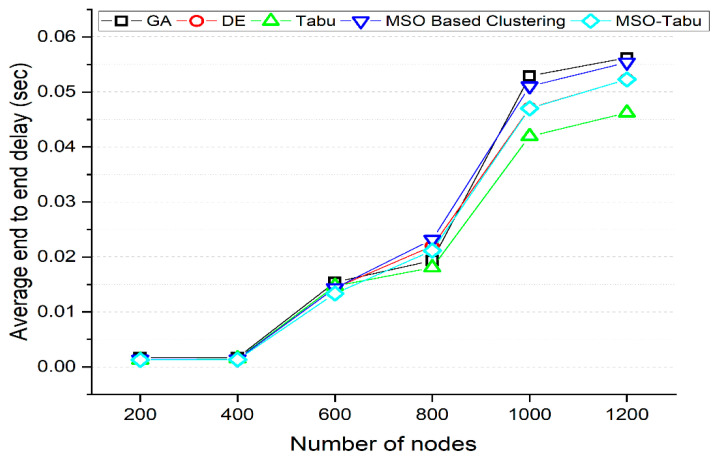
Average End to End Delay for MSO-Tabu.

**Figure 3:**
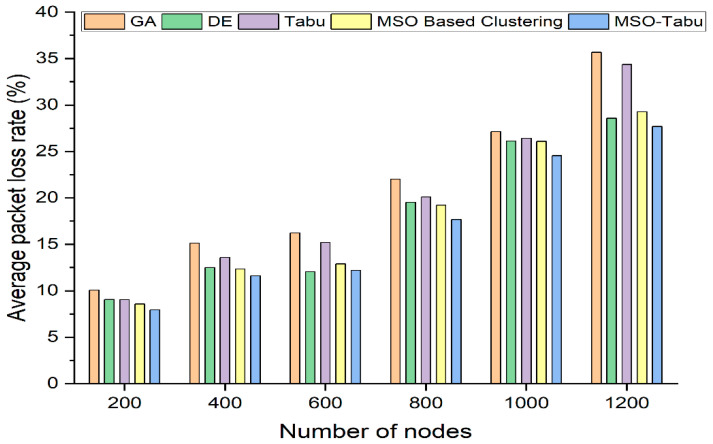
Average Packet Loss Rate for MSO-Tabu.

**Figure 4:**
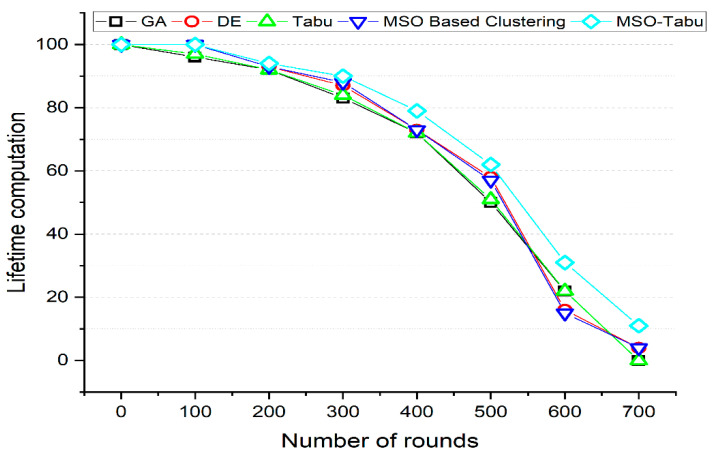
Lifetime Computation for MSO-Tabu.

**Figure 5:**
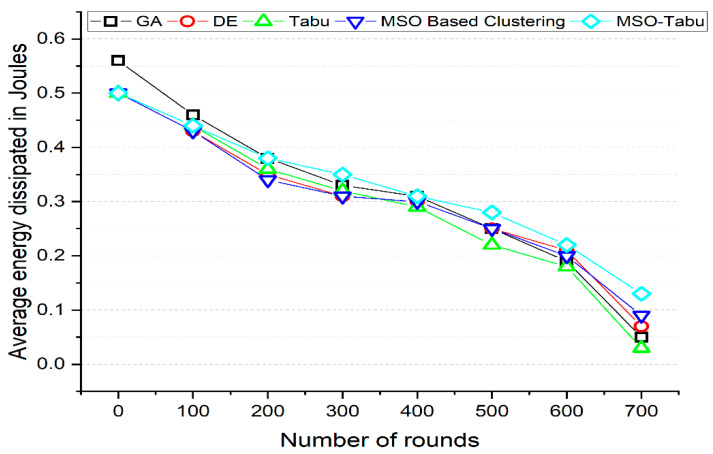
Average energy dissipated in Joules for MSO-Tabu.

The experiments are carried out using 200 to 1200 nodes and 0 to 700 rounds. The number of clusters created, average end-to-end delay in seconds, average packet loss rate, lifetime computation, and average energy dissipated in joules as shown in Table 2, Table 3, Table 4, Table 5 and Table 6 and Figure 1, Figure 2, Figure 3, Figure 4 and Figure 5.

From Figure 1, it can be observed that the MSO-Tabu has a higher number of clusters formed when compared to the other four metaheuristics. The improvement in MSO-Tabu is approximately 30%, 13%, 8%, 7%, 14%, and 11% as compared to GA for 200, 400, 600, 800, 1000, and 1200 number of nodes respectively. Similarly, as compared to DE, the advantage of MSO-Tabu is 8%, 13%, 4%, 3%, 3%, and 0%. The improvement of MSO-Tabu over Tabu is 18%, 13%, 4%, 11%, 14%, and 8% whereas over MSO based clustering the advantage is 8%, 6%, 4%, 3%, 3%, and 0% when compared for 200, 400, 600, 800, 1000, and 1200 number of nodes, respectively.

From Figure 2, it can be observed that the MSO-Tabu has a lower average end to end delay by 24%, 22%, 13%, 11%, and 7% for 200, 400, 600, 1000, and 1200 number of nodes, but higher delay by 9% for 800 number of nodes when compared with GA. It is observed that MSO-Tabu has the average end-to-end delay advantage of 0%, 4%, 7%, 4%, 0%, and 0% compared to DE. At the same time, MSO-Tabu, when compared with Tabu, has a lower average end to end delay by 0%, 11%, and 9% for 200, 400, and 600 nodes but a higher average end to end delay of 17%, 12%, and 13% for 800, 1000, and 1200 number of nodes respectively. With respect to MSO based clustering, the proposed MSO-Tabu has 8%, 6%, 7%, 9%, 8%, and 6% improvement for 200, 400, 600, 800, 1000, and 1200 number of nodes respectively. It is also observed that the delay performance characteristics of MSO-Tabu are similar to DE.

From Figure 3, it can be observed that the MSO-Tabu has a lower average packet loss rate by 21%, 23%, 25%, 20%, 9%, and 22% as compared to GA and by 12%, 14%, 20%, 12%, 7%, and 19% as compared to Tabu for 200, 400, 600, 800, 1000, and 1200 number of nodes respectively. It is also observed that MSO-Tabu perform better than MSO based clustering by 7%, 6%, 6%, 8%, 6%, and 5% for 200, 400, 600, 800, 1000, and 1200 nodes respectively. However, in the case of DE for 600 nodes, the performance of MSO-Tabu is lower than DE by 1%. Nevertheless, MSO-Tabu’s performance is better than DE by 12%, 7%, 9%, 6%, and 3% for 200, 400, 800, 1000, and 1200 number of nodes.

From Figure 4, it can be observed that the MSO-Tabu has higher lifetime computation by 0%, 4%, 2%, 8%, 10%, 24%, 41%, and 1000% for GA and by 0%, 0%, 1%, 3%, 8%, 7%, 94%, and 175% for DE when compared for 0, 100, 200, 300, 400, 500, 600, and 700 number of rounds respectively. For the same numbers of round, MSO-Tabu also reports better lifetime characteristics by 0%, 3%, 2%, 7%, 10%, 22%, 41%, and 1000% for Tabu and 0%, 0%, 1%, 2%, 8%, 9%, 107%, and 175% for MSO based clustering algorithm respectively.

From Figure 5, it can be observed that the MSO-Tabu has higher average energy dissipation by 0%, 2%, 9%, 3%, 3%, 12%, 5%, and 86% as compared to DE, by 0%, 0%, 6%, 9%, 7%, 27%, 22%, and 333% as compared to Tabu and 0%, 2%, 12%, 13%, 3%, 12%, 10%, and 44% as compared to MSO based clustering when compared for 0, 100, 200, 300, 400, 500, 600, and 700 number of rounds respectively. However, when compared with GA, the MSO-Tabu has lower average energy dissipation by 11% and 4% for 0 and 100 number of rounds, but higher average energy dissipation by 0%, 6%, 0%, 12%, 16%, and 160% for 200, 300, 400, 500, 600, and 700 number of rounds respectively. Thus, MSO-Tabu is also able to secure better energy dissipation characteristics than all the metaheuristics.

## 5. Conclusions

To operate WSNs effectively and reliably, the most important requirement is energy-saving, and for significant technical problems and resource management, much importance is presented in energy consumption. This work’s major aim is to improve the WSN CH selection through a hybrid heuristic system. Thus, an algorithm based on MSO and TS is intended to optimize the routing in WSN and CH selection. The presented method is energy efficient and in response to the network. Effective CHs are chosen by the presented technique and in response to the network. Effective CHs selected by the proposed scheme optimizes the routing and increases the network’s lifespan. Based on the exhaustive comparison of the proposed MSO-Tabu approach with GA, DE, Tabu, and MSO based clustering, the following conclusions are drawn

- In terms of the number of clusters formed, the proposed MSO-Tabu approach is on an average 14%, 5%, 11%, and 4% better than GA, DE, Tabu, and MSO based clustering, respectively.
- MSO-Tabu has lower average end-to-end delay by 11%, 2%, and 7% compared to GA, DE, and MSO based clustering, respectively. However, for the various number of nodes, the average performance of Tabu is better than MSO-Tabu by 4%.
- In terms of average packet loss rate, the proposed MSO-Tabu approach is 20%, 6%, 14%, and 6% better than GA, DE, Tabu, and MSO based clustering, respectively.
- MSO-Tabu has higher lifetime computation by 136%, 36%, 136%, and 38% compared to GA, DE, Tabu, and MSO based clustering, respectively.
- The average energy dissipated by MSO-Tabu is 22%, 16%, 51%, and 12% higher than GA, DE, Tabu, and MSO based clustering, respectively.

The key limitation of the proposed algorithm lies in tuning parameters selection, which is not adaptive and is static in nature. Noticeably, the selection of diversity control parameters has a major role in the convergence of the algorithm in the solution space. Improper selection of those parameters results in poor local search capability of the MSO-Tabu algorithm. However, the proposed algorithm provides stable performance concerning changes in other parameters under consideration.

## Figures and Tables

**Table 1:** Parameters of the network.

Parameters	Description
Transmission Range	30 m
Sensing range of node	10 m
Initial energy of node	40 Joules
Bandwidth of node	50 kbps
Size of network	300 square meters
Size of each region	75 square meters
Packet transmission rate	30 packets/seconds
Mobility model	Random waypoint
Simulation time	35 min
Tx energy	16 milliWatt
Rx energy	12 milliWatt
Power intensity	−20 dBm to 12 dBm
Mobility	0.5 m/s to 3.5 m/s
Size of packets	8, 16, 32, 64, 128, 256 bytes

**Table 2:** Number of clusters formed for MSO-Tabu.

Number of Nodes	GA	DE	Tabu	MSO Based Clustering	MSO-Tabu
200	10	12	11	12	13
400	16	16	16	17	18
600	26	27	27	27	28
800	29	30	28	30	31
1000	29	32	29	32	33
1200	35	39	36	39	39

**Table 3:** Average end-to-end delay for MSO-Tabu.

Number of Nodes	GA (s)	DE (s)	Tabu (s)	MSO Based Clustering (s)	MSO-Tabu (s)
200	0.00169	0.001284	0.001285	0.001387	0.00128
400	0.001709	0.001391	0.001513	0.001427	0.00134
600	0.015403	0.014344	0.014703	0.014344	0.01334
800	0.019371	0.021970	0.018068	0.023170	0.02115
1000	0.052919	0.047054	0.041950	0.051054	0.04701
1200	0.056208	0.052300	0.046218	0.055377	0.05228

**Table 4:** Average packet loss rate for MSO-Tabu.

Number of Nodes	GA	DE	Tabu	MSO Based Clustering	MSO-Tabu
200	10.07	9.06	9.04	8.56	7.95
400	15.1	12.51	13.6	12.32	11.63
600	16.24	12.06	15.19	12.9	12.18
800	22.01	19.51	20.09	19.2	17.67
1000	27.11	26.11	26.42	26.09	24.54
1200	35.68	28.56	34.35	29.27	27.69

**Table 5:** Lifetime computation for MSO-Tabu.

Number of Rounds	GA	DE	Tabu	MSO Based Clustering	MSO-Tabu
0	100	100	100	100	100
100	96	100	97	100	100
200	92	93	92	93	94
300	83	87	84	88	90
400	72	73	72	73	79
500	50	58	51	57	62
600	22	16	22	15	31
700	1	4	1	4	11

**Table 6:** Average energy dissipated in Joules for MSO-Tabu.

Number of Rounds	GA	DE	Tabu	MSO Based Clustering	MSO-Tabu
0	0.56	0.5	0.5	0.5	0.5
100	0.46	0.43	0.44	0.43	0.44
200	0.38	0.35	0.36	0.34	0.38
300	0.33	0.31	0.32	0.31	0.35
400	0.31	0.3	0.29	0.3	0.31
500	0.25	0.25	0.22	0.25	0.28
600	0.19	0.21	0.18	0.2	0.22
700	0.05	0.07	0.03	0.09	0.13

## Data Availability

Not applicable.
